# Supplementary material for: Hierarchical social modularity in gorillas
Source: Proc Biol Sci. 2019 Jul 10;286(1906):20190681. doi: 10.1098/rspb.2019.0681 (PMC6650716; doi:10.1098/rspb.2019.0681)
Supplement: Extended Results and Methods [file rspb20190681supp1.pdf]

Supplementary Material

Hierarchical Social Modularity in Gorillas

Robin E. Morrison[1], Milou Groenenberg[2], Thomas Breuer[2,3], Marie L. Manguette[2,4], Peter D. Walsh[5]

1. Department of Archaeology, University of Cambridge, Downing Street, Cambridge, CB2 3DZ
2. Mbeli Bai Study, Wildlife Conservation Society - Congo Program, B.P. 14537 Brazzaville, Republic of Congo
3. World Wide Fund for Nature, Reinhardtstrasse 18, 10117 Berlin, Germany
4. Max Planck Institute for Evolutionary Anthropology, Deutscher Platz 6, 04103 Leipzig, Germany
5. Apes Incorporated, 5301 Westbard Circle, Bethesda, MD 20816

Ape social trait phylogenies

Ape mating system properties show both a high degree of plasticity within species and a high level of diversity between species. However, if we focus only on the size of alliances of males cooperating to control access to females (the number of socially bonded males within a single, reproductive, social group), the pattern becomes much less plastic and diverse. These male alliance sizes also relate more generally to the origins of higher level human cooperative networks, which tend not to hinge on the pattern of mating between males and females. Single male reproductive groups are the dominant theme in gibbons (*Hylobatidae*), orang-utans (*Pongo*), gorillas (*Gorilla*), and in human hunter gather societies, with less than 10% of reproductive units involving cooperation between more than one (most often two) male (1–5). Thus, we scored all of these taxa as predominantly single male. Communities in both species of the genus *Pan* are composed of alliances of multiple cooperating males (6,7), and these were therefore scored as multi-male. Ancestral state reconstruction shows broad support for single males defending a female (or females) at all internal nodes prior to the *Pan* genus (Fig. S4). In order for early hominins to have had multi-male groups, three evolutionary transitions are required: transitions from single to multi-male in the mountain gorilla sub-species and the *Pan/Homo* common ancestor, and a reversal back to single-male groups suggested to have taken place at roughly 1myBP (8). In contrast, if the *Pan/Homo* common ancestor is reconstructed as single male, only two transitions are required: single male to multi-male in both mountain gorillas and *Pan*.

Social interaction

Simple ratio association values were calculated for interactions that took place in the bai when individuals were ≤100m apart. Association values based on same day visits were highly consistent with association values based on interactions within 100m (Mantel test:

Z=0.242 p=0.001) demonstrating that close proximity social interaction could be well predicted by visitation pattern. Modularity analysis on the social network based on interactions at ≤100m produced social units for which presence or absence of a pair of silverbacks in the same social module agreed with those based on same day visits in 290 out of 465 cases (62.24% $X^2(3, N=465) = 8.84$ p=0.0029), further demonstrating the utility of using associations based on movement patterns to predict social interaction at a close spatial scale, whilst still remaining sensitive to potential long range social interactions such as chest beating.

Observational estimates of kinship at Mbeli bai

In contrast to the results from the Lokoué dataset, observational estimates of kinship in the Mbeli population from presence in the same group prior to sexual maturity, did not predict membership of the same module using either the simple ratio index (z= 0.4, Pr(>|z|) 0.69) or the binomial probability index (z= 1.0, Pr(>|z|) 0.343). This suggests that kinship may not be driving the associations detected in the Mbeli population; however the involuntary transfer of non-adult males and mothers with offspring between groups (9), and the low sample size of adult males of known natal groups may be obscuring any potential relationship between social structure and kinship.

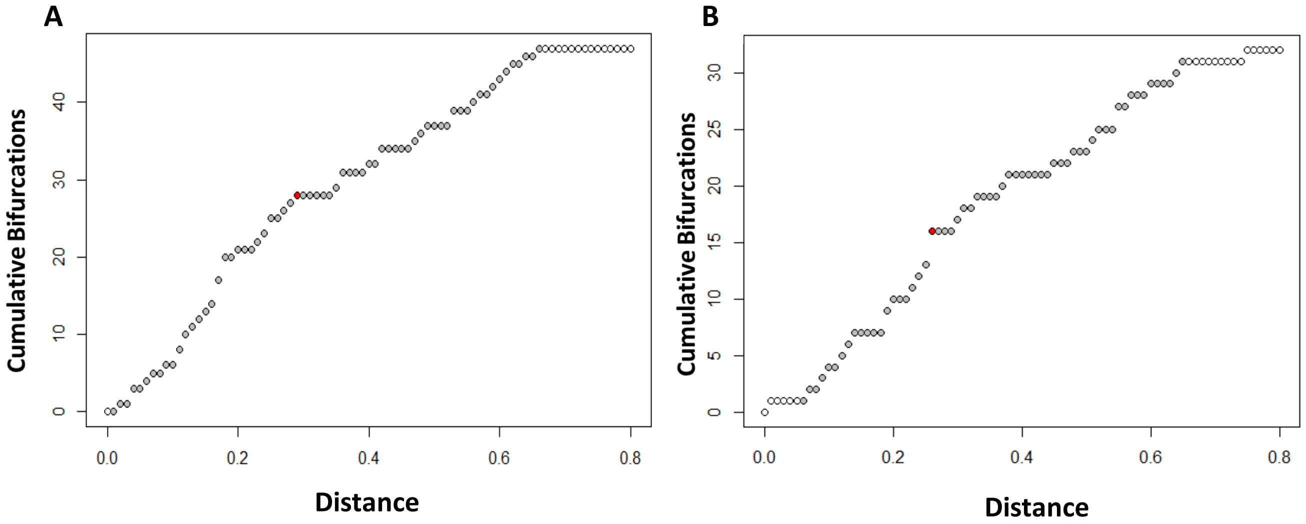

Fig. S1. Plots of cumulative bifurcations by distance with significant knots indicated in red. Lokoué (A) with knot height = 0.29 and Mbeli dataset C (B) with knot height = 0.26. Grey filled circles indicate values used in knot detection.

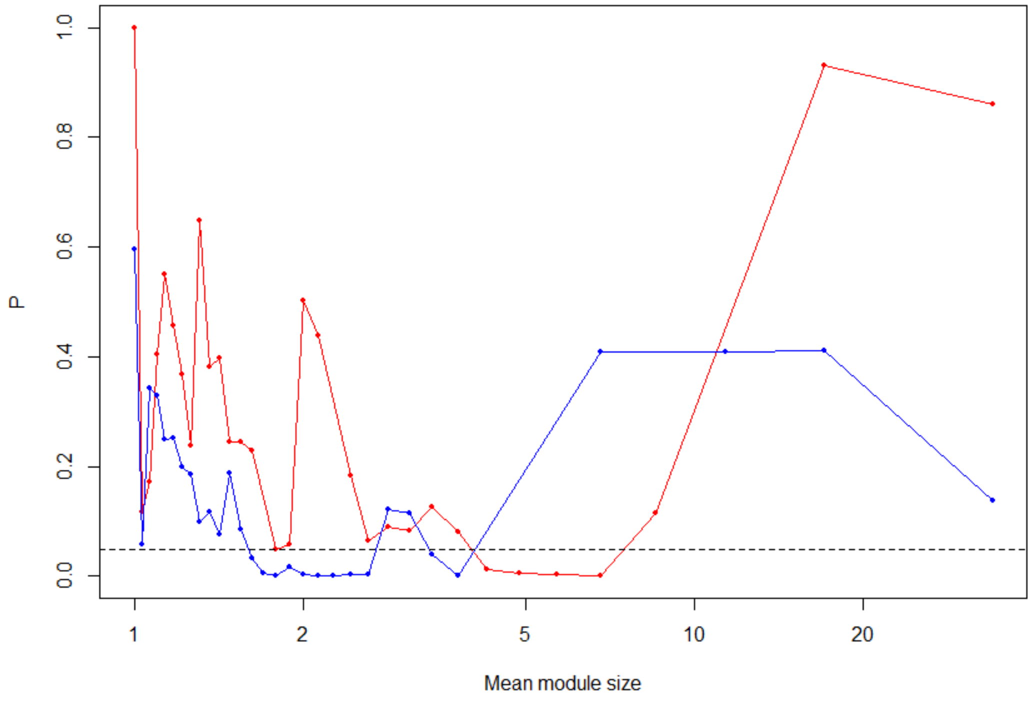

Fig. S2. Minimum P-values of modularity scores for a given size of module for Lokoué (red) and Mbeli dataset C (blue) populations, produced by varying the modularity resolution parameter.

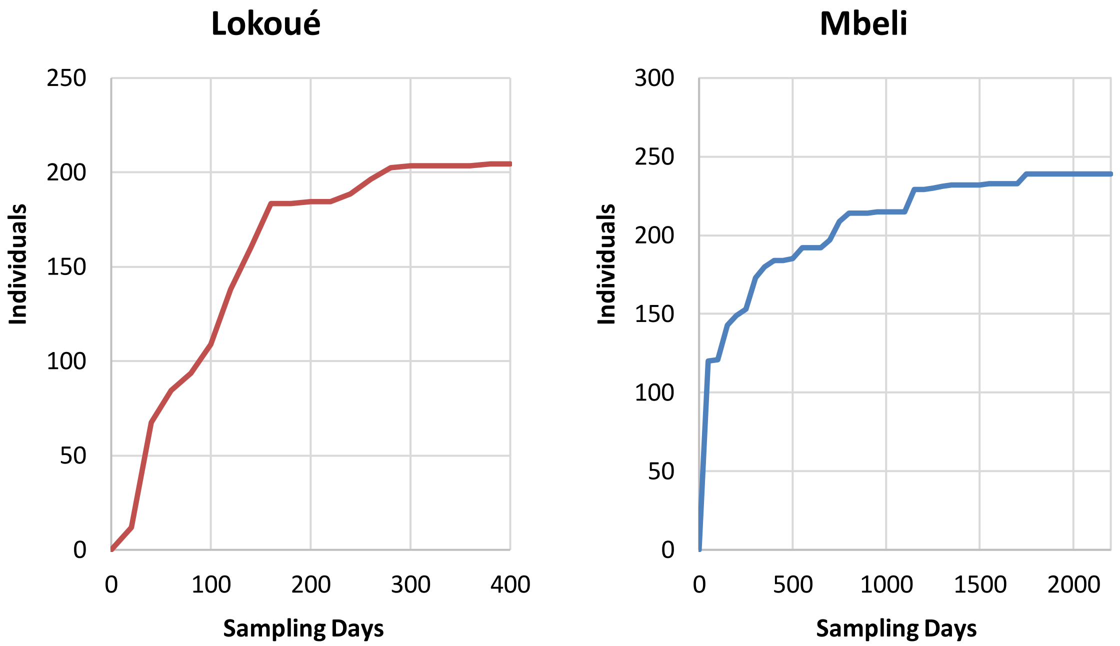

Fig. S3. Population accumulation curves for Lokoué (red) and Mbeli (blue) across the sampling periods. Cumulative number of individuals estimated as the sum of group sizes and solitaries observed in the clearing within the given number of sampling days. Community closure indicated by asymptotic shape of accumulation curves.

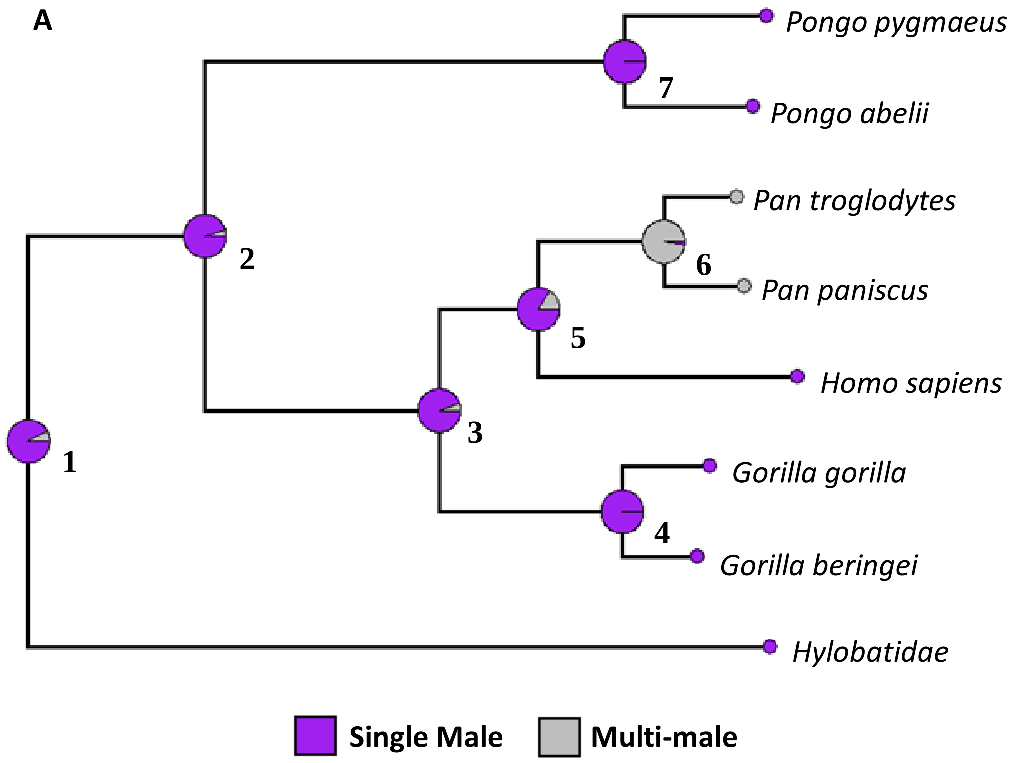

B

Node	Single Male	Multi-male
1	0.919	0.081
2	0.953	0.047
3	0.943	0.057
4	0.998	0.002
5	0.840	0.160
6	0.024	0.976
7	0.997	0.003

Fig. S4. Ancestral state reconstruction of male alliance size in apes (1–7,10), with empirical Bayesian posterior probabilities of single male alliances (purple) and multi-male alliances (grey) at each internal node (1-7) indicated by pie chart, and the trait of extant species indicated by coloured circle at tip. B) Precise values for empirical Bayesian posterior probabilities for alliance size state for internal nodes (1-7) as indicated in A.

Table S1. Population composition of datasets used in social unit scaling analyses

	Groups	Adult Females	Solitaries	Total Individuals
**Lokoué**	27	64	21	205
**Mbeli C**	24	44	9	220
**Maya Nord**	31	116	17	364

Table S2. Mean social unit size by social level across three gorilla populations (Mbeli represented using dataset C). Values for levels 4 and 5 calculated by both methods (Method A: Binomial probability index with hierarchical clustering (level 5) and modularity (level 4) analyses; Method B: Simple ratio index with varying resolution of algorithm (level 5) and modularity (level 4) analyses.

	**Social Level**			**Lokoué**	**Mbeli**	**Maya Nord**
**1**	Individual	G1		1	1	1
**2**	Mother-Offspring unit			2.45	2.67	2.72
**3**	Group	G2		6.81	8.79	11.19
**4**	dispersed extended family group	G3	Method A	9.76	12.94	*
			Method B	11.39	14.67	
**5**	aggregated group	G4	Method A	51.25	55	*
			Method B	34.17	44	
**6**	Sub-population	G5		*	*	*
**7**	Bai Population	G6		205	220	364

*unknown

Table S3. Scaling statistics across three gorilla populations (Mbeli represented using dataset C) and for all combined, using social unit sizes calculated by both methods. Method A: Binomial probability index with hierarchical clustering and modularity analyses; Method B: Simple ratio index with varying resolution of algorithm and modularity analyses.

	**Method A**			**Method B**		
	**Scaling Ratio**	**R squared**	**P**	**Scaling Ratio**	**R squared**	**P**
**Lokoué**	2.78	0.984	9.91e-05	2.47	0.996	5.71e-06
**Mbeli**	2.74	0.987	6.12e-05	2.57	0.994	1.43e-05
**Maya Nord**	3.07	0.993	0.00369	3.07	0.993	0.00369
**Combined**	2.83	0.988	2.90e-09	2.65	0.994	8.02e-11

Extended Methods

Data sets

The 4 datasets used in this research were made up of the populations listed in table S1. Maturing males were considered independent from their natal groups (solitary) from the last point at which they were observed with said groups. Only males that were independent prior to the start of the dataset study period were included in the analyses. All datasets provided binary presence/absence information for all groups and solitaries by day, which was collected by non-invasively monitoring these naturally occurring forest clearings, and individually identifying gorillas through the use of telescopes and cameras, and comparison with a database of known gorillas (9,12–14).

Binomial Probability Index (BPI)

To account for environmental effects on visit rate and reduce the influence of extreme association values calculated from units (groups or solitaries) with low numbers of observed visits, a novel association index was developed. This was based on the binomial probability of observing a pair of groups or solitaries in the clearing on the same day, more than the number of times demonstrated in the data (observed co-visits), given how often both units visited over the entire study period. The binomial probability index (BP) was calculated as the square root of the cumulative binomial probability of seeing greater than the observed number of co-visits by a pair of units, across all days in the dataset, as shown in equation 1 where *F(x)* represents the cumulative probability distribution.

(1) The binomial probability index:

$$\text{BP} = \sqrt{1 - F(x)}$$

The cumulative probability distribution was calculated using the total number of visits for each unit, whilst controlling for variation in the relative popularity of the clearing to gorillas due to potential seasonal or environmental factors, such as ripe fruits on the clearing edge (15). This was controlled for to account for the potential for such factors to lead to units encountering one another more often, driving inflated association values. Relative popularity (RP) was calculated as shown in equation 2, by summing the number of units that visited the clearing on the day in question, the five days previous, and the five days after, to produce the expected proportion of total units to visit on that day.

$$\textbf{(2)}\ \text{Relative popularity (RP)} = \frac{\text{Visit frequency in 11 day window}}{11 \times \text{Total visit frequency}}$$

The probability of a co-visit for each pair of units was calculated for each day by multiplying the number of times each unit visited the clearing over the entire period, and the relative popularity of that day. This was summed across all days to produce the expected number of co-visits across the study period for that pair (equation 3). The first 5 days and last 5 days were not included in these analyses.

$$\textbf{(3)}\ \text{Expected co-visits} = \sum \text{RP}^2 \times \text{V}_\text{a} \times \text{V}_\text{b}$$

Where Va = Total visits by group A
and Vb = Total visits by group B

This expected co-visits value, divided by the total number of days was used as the mean probability of a co-visit within the binomial formula. F(x) was calculated using the 'pbinom' function in R, with observed number of co-visits, mean probability of a co-visit, and the total days within the dataset, as input values.

This index was unable to account for variation in individual home ranges, as ranging patterns of gorillas outside the forest clearings were largely unknown. Therefore environmental conditions in home ranges could potentially influence the gorilla association patterns detected. However this should be minimised by the large sample sizes of individual gorillas in the datasets, and the considerable time periods covered. Furthermore, such an environmental driver to association patterns would not make the associations themselves any less valid. Human social networks are well predicted by spatial overlap, with close spatial proximity both increasing the likelihood of new social ties forming, and decreasing the likelihood of those social ties breaking down(16). If high range overlap increased the likelihood of contact between gorillas, this could in itself lead to closer social affiliations developing. Null models were created by generating data sets where the presence of a unit on a specific day was determined by a random probability (random number generation between 0 and 1 under a uniform distribution) in combination with their visit rate over all days and the visit rate of all gorillas on that day. BP association index matrices were then calculated for all 1000 random datasets using the same method as the observed data, as discussed above.

Hierarchical clustering

The region of distances for which gradient changes were investigated were specified as distance values above which >50% of random models already had an initial bifurcation, and values below which <50% had already fully bifurcated. R^2 values were calculated to identify how well the data were predicted by linear models. Changes in gradient in the observed datasets were then identified using a Wilcoxon Two Sample Test, for each distance datapoint, and that with the lowest P-value selected as the knot. As plotting the cumulative number of bifurcations with height did not show a clear linear relationship in the simple ratio null models, despite various data transformations, only association matrices produced using the binomial probability index were investigated using this method.

Varying modularity

As the Lokoué population had such strong modularity ($p<0.001$) for the majority of resolution values, the sample size was reduced from 48 silverbacks to 34, by including only those that visited at least 10 times during the study period (previously 8). This enabled clear variation in modularity P-values with resolution (and therefore social unit size).

Social interaction

Simple ratio association values were calculated using intergroup (or solitary) interactions that took place in the bai when individuals were ≤100m apart during 2015 and 2016. Consistency between these association indices and those calculated from same day visits of groups was investigated using a Mantel test. Social modules from intergroup interactions (at ≤100m) were identified using the Louvain multi-level modularity optimisation algorithm (17) and compared with those identified from same day visit data when using only groups and

solitaries for which intergroup interaction data was available. Agreement in presence/absence of silverbacks in the same module between both methods was tested using chi squared.

Scaling

Scaling of social unit size was investigated using results from the Lokoué and Mbeli C datasets, as well as additional data from Maya-Nord(18), a further western gorilla study site. The total individuals included all gorillas identified in each bai during the dataset period. Mean mother-offspring unit size was calculated by equation (3). Mean group size was calculated by equation (4). Sizes of above-group social units were calculated by equation (5).

$$(1)\ \text{Mean mother offspring unit size} = \frac{\text{total individuals – adult male}}{adult\ females}$$

$$(2)\ \text{Mean group size} = \frac{\text{total individuals – solitary males}}{number\ of\ groups}$$

$$(3)\ \text{Mean above-group social unit size} = \frac{\text{total individuals}}{number\ of social\ units}$$

Population accumulation curves were plotted as the number of individuals observed as the sampling period progressed. Group sizes were those reported for the Lokoué dataset. For Mbeli, group sizes were estimated as the maximum number of individuals observed in the group, present in the bai during the two year period. The mean across all periods for which each group was present was then used as an estimate for overall group size. The cumulative number of individuals was then estimated as the sum of the group sizes and solitaries for those that had been observed in the bai within the given number of sampling days.

Kinship

Published kinship data from Lokoué was binary, with 1 indicating an estimated relatedness of ≥0.2 and 0 indicating an estimated relatedness of <0.2. This cut-off should assign all pairs that are half-siblings (relatedness=0.25) or more closely related, a value of 1, with some room for error in estimate precision. Pairwise relatedness estimates from long term behavioural observations (presence in the same group prior to sexual maturity) from the Mbeli population (n=16) were used to predict co-membership of the same social unit using binomial logistic regression. Presence in the same group prior to sexual maturity from the behavioural data at Mbeli Bai should be consistent with Lokoué genetic data as individuals would be expected to share at least one parent and therefore be (at least) half siblings if they grew up in the same group, however extra-group matings and migration into new groups prior to sexual maturity could considerably reduce the accuracy of this estimate. Social units were identified as those detected by modularity analysis using both association index measures. Association type (group-group, solitary-group or solitary-solitary) was included in the regressions to control for differences in interactions between solitaries and groups. The kinship of silverbacks in the smaller social units could not be investigated due to the low sample of pairs in the same module for which relatedness was known.

Ape phylogenies and ancestral state reconstruction

Male alliance size states were reconstructed for ancestral nodes across a great ape phylogenetic tree from the 10k trees project (11). Properties in extant taxa at the species level were determined through a thorough review of the literature. Ancestral state reconstruction was carried out using the 'ace' function from the 'ape' R package (19) using a

continuous-time Markov chain model. Traits were fitted with an equal-rates model ("ER"), due to the relatively small number of species present in the phylogeny and therefore the lack of adequate transitions to accurately estimate different rates of transition between different states. The presence or absence of HSM was plotted on the same ape phylogenetic tree; however ancestral state reconstruction was not attempted for this trait due to the lack of knowledge about the presence of HSM in most ape species.

References

1. Fuentes A. Hylobatid communities: Changing views on pair bonding and social organization in hominoids. Am J Phys Anthropol. 2000;43:33–60.

2. Singleton I, Van Schaik CP. The social organisation of a population of Sumatran orang-utans. Folia Primatologica. 2002. p. 1–20.

3. Yamagiwa J. Diachronic changes in two eastern lowland gorilla groups (Gorilla gorilla graueri) in the Mt. Kahuzi Region, Zaïre. Primates. 1983;24(2):174–83.

4. Walker RS, Hill KR, Flinn M V., Ellsworth RM. Evolutionary history of hunter-gatherer marriage practices. PLoS One. 2011;6(4):2–7.

5. Breuer T, Robbins AM, Olejniczak C, Parnell RJ, Stokes EJ, Robbins MM. Variance in the male reproductive success of western gorillas: Acquiring females is just the beginning. Behav Ecol Sociobiol. 2010;64(4):1–14.

6. Kalpers J, Williamson EA, Robbins MM, McNeilage A, Nzamurambaho A, Lola N, et al. Gorillas in the crossfire: Population dynamics of the Virunga mountain gorillas over the past three decades. ORYX. 2003;37(3):326–37.

7. Stanford CB. The Social Behavior of Chimpanzees and Bonobos: Empirical Evidence and Shifting Assumptions. Curr Anthropol. 1998;39(4):399–420.

8. Foley R, Gamble C. The ecology of social transitions in human evolution. Philos Trans R Soc Lond B Biol Sci. 2009;364(1533):3267–79.

9. Stokes EJ, Parnell RJ, Olejniczak C. Female dispersal and reproductive success in wild western lowland gorillas (Gorilla gorilla gorilla). Behav Ecol Sociobiol. 2003;54(4):329–39.

10. Yamagiwa J, Kahekwa J, Basabose AK. Intra-specific variation in social organization of gorillas: Implications for their social evolution. Primates. 2003;44(4):359–69.

11. Arnold C, Matthews LJ, Nunn CL. The 10kTrees website: A new online resource for primate phylogeny. Evol Anthropol. 2010;19(3):114–8.

12. Parnell RJ. The social structure and behaviour of western lowland gorillas (Gorilla gorilla gorilla) at Mbeli Bai, Republic of Congo. Dr Diss Stirling. 2002;

13. Levréro F. Structure d'une population de gorilles (Gorilla g. gorilla) visitant une clairière forestière: nature et rôle des rencontres intergroupes dans sa dynamique. Dr Diss Rennes. 2005;

14. Breuer T, Hockemba MBN, Olejniczak C, Parnell RJ, Stokes EJ. Physical maturation, life-history classes and age estimates of free-ranging western gorillas - Insights from Mbeli Bai, Republic of Congo. Am J Primatol. 2009;71(2):106–19.

15. Walsh PD, Breuer T, Sanz C, Morgan D, Doran-Sheehy D. Potential for Ebola

Transmission between Gorilla and Chimpanzee Social Groups. Am Nat. 2007;169(5):684–9.

16. Rivera MT, Soderstrom SB, Uzzi B. Dynamics of Dyads in Social Networks: Assortative, Relational, and Proximity Mechanisms. Annu Rev Sociol. 2010;36(1):91–115.

17. Blondel VD, Guillaume J-L, Lambiotte R, Lefebvre E. Fast unfolding of communities in large networks. J Stat Mech. 2008;

18. Magliocca F, Querouil S, Gautier-Hion a. Population structure and group composition of western lowland gorillas in north-western Republic of Congo. Am J Primatol. 1999;48(1):1–14.

19. Paradis E, Schliep K. ape 5.0: an environment for modern phylogenetics and evolutionary analyses in R. Bioinformatics. 2019;35(3):526–8.
